# Functioning and health in patients with cancer on home-parenteral nutrition: a qualitative study

**DOI:** 10.1186/1477-7525-8-41

**Published:** 2010-04-16

**Authors:** Martin Mueller, Stefanie Lohmann, Paul Thul, Arved Weimann, Eva Grill

**Affiliations:** 1 Institute for Health and Rehabilitation Sciences, Ludwig-Maximilians-University, Munich, Germany; 2 ICF Research Branch of WHO FIC CC (DIMDI) at SPF Nottwil, Switzerland, and at IHRS, Ludwig-Maximilians-University, Munich, Germany; 3 Department of General, Visceral, Vascular and Thoracic Surgery, Charité Campus Mitte, Humboldt-University, Berlin, Germany; 4 Clinic for General and Visceral Surgery, Klinikum St. Georg, Leipzig, Germany

## Abstract

**Background:**

Malnutrition is a common problem in patients with cancer. One possible strategy to prevent malnutrition and further deterioration is to administer home-parenteral nutrition (HPN). While the effect on survival is still not clear, HPN presumably improves functioning and quality of life. Thus, patients' experiences concerning functioning and quality of life need to be considered when deciding on the provision of HPN. Currently used quality of life measures hardly reflect patients' perspectives and experiences. The objective of our study was to investigate the perspectives of patients with cancer on their experience of functioning and health in relation to HPN in order to get an item pool to develop a comprehensive measure to assess the impact of HPN in this population.

**Methods:**

We conducted a series of qualitative semi-structured interviews. The interviews were analysed to identify categories of the International Classification of Functioning, Disability and Health (ICF) addressed by patients' statements. Patients were consecutively included in the study until an additional patient did not yield any new information.

**Results:**

We extracted 94 different ICF-categories from 16 interviews representing patient-relevant aspects of functioning and health (32 categories from the ICF component 'Body Functions', 10 from 'Body Structures', 32 from 'Activities & Participation', 18 from 'Environmental Factors'). About 8% of the concepts derived from the interviews could not be linked to specific ICF categories because they were either too general, disease-specific or pertained to 'Personal Factors'. Patients referred to 22 different aspects of functioning improving due to HPN; mainly activities of daily living, mobility, sleep and emotional functions.

**Conclusions:**

The ICF proved to be a satisfactory framework to standardize the response of patients with cancer on HPN. For most aspects reported by the patients, a matching concept and ICF category could be found. The development of categories of the component 'Personal Factors' should be promoted to close the existing gap when analyzing interviews using the ICF. The identification and standardization of concepts derived from individual interviews was the first step towards creating new measures based on patients' preferences and experiences which both catch the most relevant aspects of functioning and are sensitive enough to monitor change associated to an intervention such as HPN in a vulnerable population with cancer.

## Background

Weight loss is a common and serious problem in patients with cancer [1-3]. In patients with cancer in the abdominal cavity weight loss is often caused by symptoms preventing sufficient food intake or digestion, e.g. bowel obstruction, fistulas or short bowel syndrome [4]. More prominently, weight loss in advanced cancer is frequently related to the anorexia-cachexia syndrome. This includes various metabolic changes leading to a waste of adipose tissue and skeletal muscle mass related to tumour progression [5,6]. In addition, side effects of antineoplastic therapy result in diminished food intake and progressive deterioration of patients' condition [7].

Malnutrition leads to physical weakness, psychological imbalances and fatigue. It not only compromises patients' functioning and hence quality of life but has also negative effects on prognosis [8]. One possible strategy to prevent malnutrition and further deterioration of functioning is to maintain sufficient caloric intake by parenteral nutrition. This can even be administered at home. Although there are some studies showing the benefits of home-parenteral nutrition (HPN) in cancer-associated malnutrition, its use is discussed controversially from both an economical and ethical position [4,9-11].

The effects of HPN on survival are well known [4]. Health-related quality of life is another relevant outcome of HPN for patients with advanced cancer [4]. Studies on quality of life, however, are inconclusive [11-13]. Although HPN potentially improves patients' functional status, performance, and participation, established quality of life measures do not capture the salient aspects relevant in this population [14,15]. This is why an instrument more specific to the effects of HPN therapy in patients with cancer is required [16]. Moreover, it is not known which issues are most relevant to those patients, and which of these issues are prone to change by the administration of HPN. Concepts used so far in the assessment of quality of life in patients on HPN lack a comprehensive theoretical framework that justifies the choice of specifically addressed items.

**Figure 1:**
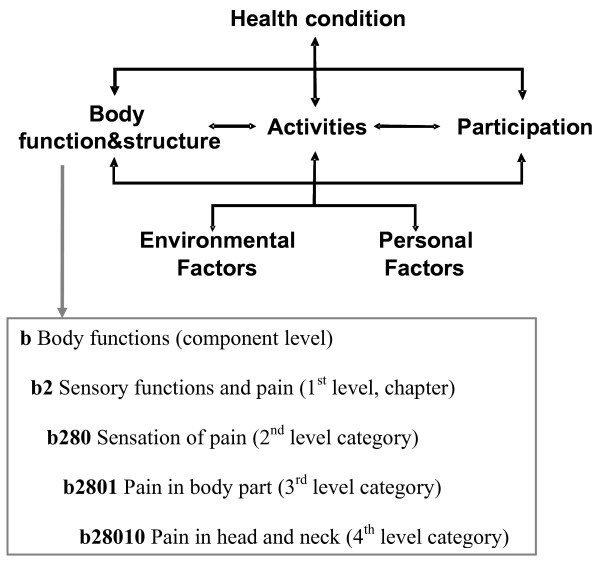
**The ICF model of functioning, disability and health and an example of the hierarchical structure of the ICF**.

The International Classification of Functioning, Disability and Health (ICF) potentially is a comprehensive and commonly accepted framework that covers the experience of human functioning as a whole [17]. The ICF is part of the WHO family of international classifications. It is both a model and a classification. The ICF model consists of two parts: Part one, referred to as 'Functioning and Disability' covers the components 'Body Functions', 'Body Structures' and 'Activities and Participation'. Part two, referred to as 'Contextual Factors' covers the components 'Environmental Factors' and 'Personal Factors' (see Figure 1). Each component consists of several 'chapters', the components Body Functions and Activities and Participation are grouped in 'blocks' additionally. The ICF model describes the individuals' functioning as a complex interaction between a health condition and contextual factors.

The ICF classification contains more than 1400 hierarchically organized categories which describe the components of the ICF model in detail up to four levels (see also Figure 1). The intention of the ICF is to record and organize a wide range of information about health and health-related states for individuals and populations. For the purpose of defining the contents of a comprehensive assessment, the ICF provides a universal language intended to be equally used and understood by health professionals and patients. Thus, it can be used to organize and standardize issues most relevant for patients with cancer on HPN while respecting patients' perspective and experiences.

The objective of our study was to investigate the perspectives of patients with cancer on their experience of functioning and health in relation to HPN in order to get an item pool to develop a comprehensive measure to assess the impact of HPN in this population. Specific aims were

(1) to identify relevant aspects of functioning and health expressed by ICF categories in those patients

(2) to explore their experiences on improvements in functioning and health due to HPN and

(3) to explore and to compare the experiences of patients shortly after the beginning of HPN in contrast to those with longer established HPN.

## Methods

### Study design

We conducted a multi-stage series of qualitative, semi-structured, face-to-face interviews using a descriptive approach [18]. The interviews were audio-recorded and transcribed verbatim.

Two different stages were chosen to address the presumably different experiences of patients in different situations: In the first stage, we included patients shortly after the beginning of HPN who are confronted with the challenge of a new therapy to cover their specific experiences with and expectations on HPN. In the second stage we included patients with established HPN who are familiar with this therapy and faced with effects of longer HPN to validate the first stage findings and to specifically explore the consequences and experiences in the situation of prolonged HPN.

### Interview guide

The interview guideline was adopted from earlier focus group and individual interview studies with the focus to explore relevant aspects of functioning and health in different populations [19,20] (see additional file 1). It was designed to address the components of the International Classification of Functioning, Disability and Health (ICF). The interview questions tackled each of the three functioning and disability components, 'Body Functions', 'Body Structures', 'Activities and Participation', and the contextual factors 'Environmental Factors' and 'Personal Factors'.

### Additionally collected data

We collected sociodemographic and disease-specific data (age, sex, living situation, site of primary tumor and duration of HPN). Additionally, to describe an overall view of functioning, the patients were asked to appraise their personal limitations in overall functioning using a horizontal visual analogue scale, ranging from zero, for complete limitation in all aspects of functioning to ten, for no limitation in functioning.

### Participants

Patients with malignant tumors undergoing HPN were recruited from a customer database of a cooperating home care provider. Potential participants were consecutively contacted and asked for their willingness to contribute to a study by their nutrition nurse. In case of preliminary consent, the patients were provided with detailed information about the study. Informed written consent had to be signed prior to the beginning of the interview.

Inclusion criteria for both stages were over 18 years of age and adequate command of the German language. Additional inclusion criterion for stage 1 was that HPN had been administered at least seven and up to 20 days. Additional inclusion criterion for stage 2 was that HPN had been administered at least for 6 weeks or was currently suspended due to stable general condition. Positive vote of the ethics committee of the Medical Faculty of Ludwig-Maximilians-University Munich was obtained prior to start.

### Data analysis

#### Qualitative Data Analysis

The Meaning Condensation Procedure [21] was used for the analysis of data content. In the first step, the verbatim transliterated transcripts of the interviews were read through to get an overview over the collected data. In the second step, the text was divided into units of meaning and the theme that dominated a meaning unit was determined. A meaning unit was defined as a specific unit of text either a few words or a few sentences with a common theme. Therefore, a meaning unit division did not follow linguistic grammatical rules. Rather, the text was divided where the researcher discerned a shift in meaning. In the third step, the concepts contained in the meaning units were identified. A meaning unit could contain more than one concept. For quality assurance reasons, the qualitative data analysis was conducted independently by two health professionals trained in the methodology (MM, SL). The results were compared and discussed prior to further analysis.

#### Linking to the ICF

The identified concepts were linked to the categories of the ICF by two health professionals (MM, SL) based on established linking rules which enable linking concepts to ICF categories in a systematic and standardized way [22]. According to these linking rules, health professionals trained in the ICF are advised to attribute each concept to the ICF category representing this concept most precisely. One concept can be linked to one or more ICF categories, depending on the number of themes contained in the concept. Consensus between the two health professionals was required to decide which ICF category should be linked to each identified concept. In case of a disagreement, a third person trained in the linking rules was consulted. In a discussion led by the third person, the two health professionals that linked the concepts stated their pros and cons for the linking of the concept under question to a specific ICF category. Based on these statements, the third person made an informed decision. For feasibility reasons, the linking procedure was restricted to the second level of the ICF. See Table 1 for a scheme of qualitative data analysis and linking.

**Table 1:** Scheme of qualitative data analysis and linking.

Interview text	Meaning unit	ICF category
"One of my problems is that I can hardly **concentrate on the things **I do (...)."	restrictions in concentrating on things	b140 Attention functions
"I had to **quit hiking **and **cycling **(...)"	quitting hiking quitting cycling	d920 Recreation and leisure (incl. *d9201 Sports*) d475 Driving (incl. *d4750 Driving human-powerded transportation*)

### Sample size

The sample size was determined by saturation. Saturation refers to the point at which an investigator has obtained sufficient information from the field [23]. In this study, we defined saturation as the point during data collection and analysis when an interview revealed less than 5% additional second level ICF categories. This strategy aims to assure maximum sensitivity to gather a maximum variety of experiences and expectations from the participants.

## Results

We conducted sixteen individual interviews from June 2007 until February 2008 (Eleven in stage 1, five in stage 2). Ten participants were female; age ranged from 33 to 83 years (median 58.5). All participants were living in a household together with family or partner. Primary tumor sites were gastric, colorectal, liver, ovarian, breast, and oral cancer. The participants in stage 1 received HPN from eight to 19 days. Participants in stage 2 received HPN from 85 days to three and a half years. Participants rated their overall functioning from 3 to 8 (median 5).

A total of 471 different meaningful concepts were extracted from the interviews (272 in stage 1, 199 in stage 2). Those 471 identified different concepts were linked to 94 different ICF-categories. Thirty-nine concepts could not be linked to specific ICF categories.

Seventy-one different ICF categories were identified as relevant aspects of functioning in patients shortly after the beginning of HPN (stage 1). Twenty-five of those ICF categories belonged to the component 'Body functions', 25 to the component 'Activity and Participation', 8 to the component 'Body Structures' and 14 to the component 'Environmental Factors'.

Fifty-nine different ICF categories were identified as relevant aspects of functioning in patients with long-time established or currently stopped HPN (stage 2). Eighteen of those ICF categories belonged to the component 'Body Functions', 24 to the component 'Activity and Participation', 5 to the component 'Body Structures' and 12 to the component environmental factors (see Table 2, Table 3, Table 4, Table 5).

**Table 2:** ICF categories relevant in patients undergoing HPN (ICF component body functions).

*ICF block or chapter* 2^nd ^level ICF category		Stage1	expected improvement	Stage2	improvement
	***Global mental functions***				
b110	Consciousness functions	x			
b126	Temperament and personality functions	x	x		
b130	Energy and drive functions	x	x	x	x
b134	Sleep functions	x	x	x	x
	***Specific mental functions***				
b140	Attention functions	x			
b144	Memory functions			x	
b152	Emotional functions	x	x	x	
b156	Perceptual functions			x	
b167	Mental functions of language			x	
b180	Experience of self and time functions			x	
	***Additional sensory functions***				
b265	Touch function			x	
b270	Sensory functions related to temperature and other stimuli	x		x	
	***Pain***				
b280	Sensation of pain	x		x	
	***Voice and speech function***				
b310	Voice functions	x			
b320	Articulation functions	x			
b330	Fluency and rhythm of speech functions	x			
	***Functions of the cardiovascular system***				
b410	Heart functions			x	
b420	Blood pressure functions			x	
	***Additional functions and sensations of the cardiovascular and respiratory systems***				
b450	Additional respiratory functions	x			
b455	Exercise tolerance functions	x	x	x	x
	***Functions related to the digestive system***				
b510	Ingestion functions	x			
b515	Digestive functions	x		x	
b525	Defecation functions	x	x		
b530	Weight maintenance functions	x	x	x	x
b535	Sensations associated with the digestive system	x			
	***Functions related to metabolism and endocrine system***				
b545	Water, mineral and electrolyte balance functions	x	x		
	***Urinary functions***				
b620	Urination functions	x			
	***Functions of the joint and bone***				
b710	Mobility of joint functions			x	
b715	Stability of joint functions	x			
	***Musle functions***				
b730	Muscle power functions	x	x	x	x
b740	Muscle endurance functions			x	
	***Movement function***				
b765	Involuntary movement functions	x			
	***Functions of the skin***				
b820	Repair functions of the skin	x			
b830	Other functions of the skin	x			

**Table 3:** ICF categories relevant in patients undergoing HPN (ICF component activities and participation).

*ICF block or chapter* 2^nd ^level ICF category		Stage1	expected improvement	Stage2	experienced improvement
	***Applying knowledge***				
d166	Reading	x			
	***General tasks and demands***				
d230	Carrying out daily routine	x	x	x	
d240	Handling stress and other psychological demands	x		x	
	***Conversation and use of communication devices and techniques***				
d350	Conversation	x			
	***Changing and maintaining body position***				
d410	Changing basic body position	x			
d415	Maintaining a body position	x	x	x	
	***Carrying moving and handling objects***				
d430	Lifting and carrying objects			x	
d440	Fine hand use	x		x	
	***Walking and moving***				
d450	Walking	x	x	x	x
d455	Moving around	x	x	x	
d460	Moving around in different locations	x		x	
d465	Moving around using equipment	x			
	***Moving around using transportation***				
d475	Driving	x			
d510	Washing oneself	x	x	x	
d520	Caring for body parts	x		x	
	***Self-care***				
d530	Toileting			x	
d550	Eating			x	x
d560	Drinking			x	x
d570	Looking after one's health	x	x		
	***Acquisition of necessities***				
d620	Acquisition of goods and services			x	
	***Household tasks***				
d630	Preparing meals	x		x	
d640	Doing housework	x	x	x	
	***Caring for household objects and assisting others***				
d650	Caring for household objects	x		x	
	***General interpersonal interactions***				
d720	Complex interpersonal interactions	x			
	***Particular interpersonal interactions***				
d750	Informal social relationships	x		x	
d760	Family relationships	x		x	
d770	Intimate relationships			x	
	***Work and employment***				
d845	Acquiring, keeping and terminating a job	x		x	x
d850	Remunerative employment			x	x
d870	Economic self-sufficiency			x	
	***Community, social and civic life***				
d910	Community life	x			
d920	Recreation and leisure	x		x	

**Table 4:** ICF categories relevant in patients undergoing HPN (ICF component body structures).

*ICF block or chapter* 2^nd ^level ICF category		Stage1	expected improvement	Stage2	improvement
	***Structures involved in voice and speech***				
s320	Structure of mouth	x			
	***Structures of the cardiovascular, immunological and respiratory systems***				
s430	Structure of respiratory system	x			
	***Structures related to digestive, metabolic and endocrine systems***				
s530	Structure of stomach	x		x	x
s540	Structure of intestine	x		x	
s550	Structure of pancreas	x			
s560	Structure of liver	x		x	
	***Structures related to movement***				
s750	Structure of lower extremity	x			
s760	Structure of trunk	x			
	***Skin and related structures***				
s830	Structure of nails			x	
s840	Structure of hair			x	

**Table 5:** ICF categories relevant in patients undergoing HPN (ICF component environmental factors).

*ICF block or chapter* 2^nd ^level ICF category		Stage1	expected improvement	Stage2	improvement
	***Products and technology***				
e110	Products or substances for personal consumption	x	x	x	
e120	Products and technology for personal indoor and outdoor mobility and transportation			x	
e155	Design, construction and building prod. and technology of buildings for private use			x	
	***Support and relationships***				
e310	Immediate family	x		x	
e315	Extended family	x			
e320	Friends	x			
e325	Acquaintances, peers, colleagues, neighbours and community members	x		x	
e330	People in positions of authority	x		x	
e350	Domesticated animals			x	
e355	Health professionals	x			
	***Attitudes***				
e410	Individual attitudes of immediate family members	x		x	
e415	Individual attitudes of extended family members	x			
e420	Individual attitudes of friends	x		x	
e425	Individual attitudes of acquaintances, peers, colleagues, neighbours and community members	x			
e430	Individual attitudes of people in positions of authority	x		x	
e445	Individual attitudes of strangers			x	
	***Systems, services and policies***				
e570	Social security services, systems and policies	x			
e580	Health services, systems and policies	x		x	

Patients in stage 1 specified expected improvement in functioning and health which corresponded to 17 different ICF-categories. Patients in stage 2 specified experienced improvements in 11 different ICF categories (see Tables 2, 3, 4, 5).

There were 39 concepts (8% of all extracted concepts) which could not be linked to specific ICF categories. Most of them (28 concepts, 6%) could not be linked to the ICF because they were too general to be linked to specific ICF categories (aspects related to mental or general health, or quality of life) or were disease-specific and thus not covered by the ICF. A smaller proportion (11 concepts, 2%) pertained to personal factors. Specifically, those concepts were "impatience or patience", "remaining/loss of sense of humor", "faith in god", "coping with illness", "personal attitude towards disease" and "struggling with anticipated death".

## Discussion

To our knowledge, this is the first study to investigate patients' perspectives on functioning and health in patients undergoing home-parenteral nutrition with the help of a comprehensive classification, the International Classification of Functioning, Disability and Health. Patients reported various aspects of functioning as relevant. Reported issues differed between patients with short-term HPN and long-term HPN. A part of those aspects of functioning was expected and experienced to improve during HPN.

Functioning is increasingly perceived as an important outcome when examining patients undergoing HPN. To give an example, the Karnofsky Performance Status Scale [24] is one of the most frequently used outcome measures [4], assessing different performance levels. Nevertheless, it does not discriminate among specific aspects of functioning. In our study, patients were able to give a very conclusive and comprehensive picture of their specific impairment and limitations when confronted with the framework of the ICF. Relevant concepts could easily be extracted from the interviews.

### Perceived limitations in Functioning and Health

Categories from all chapters of the ICF component 'Body Functions' were represented. Patients reported impairments in mental and sensory functions referring to general symptoms of malignant disease such as pain, disturbed sleep, changes in temperament and emotional functions or diminished attention [25-27]. Other impairments associated with antineoplastic therapy, e.g. impairment of sensory functions or problems with functions of the skin and hair, [28-30] were mentioned. Patients reported consequences of malnutrition such as decreased muscle power and muscle endurance, and impaired exercise tolerance. Problems with fluid and caloric intake were also reported, resulting in disturbed metabolic, endocrine and urinary functions. This is in line with literature describing functional consequences of malignancy and subsequent therapy [31,32]. Persoon et al. [14] reported similar symptoms in a population of patients with long-term HPN including patients with non-malignant disease. Limitations in functions related to the cardiovascular und respiratory system are also well known as general symptoms of malignant disease [33,34].

Of the ICF component 'Body Structures', most of the specified categories corresponded to the sites of malignancy. Also, patients at stage 2 of the interviews reported impaired structures of hair and nails, corresponding to side effects of radiation or chemotherapy [28,29]. One patient reported impairment of 'Structure of the lower extremity' which were not site of malignancy:

"Everything is okay except for the function of my right leg (...). They took a piece from there and put it into my jaw. Now I have a 20 to 25 cm long scar. They took a piece of my bone...hip bone together with tissue, muscle tissue(...)."

Since the sites of malignance differ from patient to patient, no univocal picture of the typically involved body structures could be drawn.

As for the ICF component 'Activities and Participation,' categories from all chapters were represented. Patients reported limitations in mobility, self-care and domestic life, aspects of transfer and moving around, and aspects of family life and social relationships. This is in line with the findings of Helbostad and colleagues, who identified mobility and self-care as most relevant for patients with advanced cancer [35]. Carrying out household tasks, and mobility are other activities frequently limited [13]. Family and social life is burdened by malignancy [36]. Although studies show that awareness of diagnosis and its consequences is not associated with time since diagnosis [37], our findings indicate that patients at stage 1 were more concerned with the immediate impacts of disease whereas patients at stage 2 were also aware of the consequences on work and employment. Another notable finding within the 'Activities and Participation'-component is that patients in stage 1 did not consider eating and drinking as relevant, whereas patients in stage 2 did.

Of the ICF component 'Environmental Factors', products and technology, as well as personal relationships and attitudes, were reported to have an impact on functioning and health. The ICF category 'Products and technology for personal consumption' covers food and drugs as well as their adverse effects. The influence of social support, both from the family, colleagues or friends is a main factor in the perception of malignant disease and can either worsen or ameliorate patients situation [38]. Equally, social security and the health care system do influence patients' functioning.

### Expected and experienced improvements in functioning and health

We could show differences between stage 1 and 2 in terms of experienced impairment and limitation. Patients at stage 2 but not at stage 1 reported limitations in specific mental functions, such as memory, emotional and perceptual functions. These limitations might have been there even in stage 1 but were probably veiled by more acute needs. Expected and experienced improvements within the component Body Functions were congruent. A benefit in weight maintenance is one of the primary goals in HPN [13,39]. Although some studies report HPN to disturb sleep [40], the patients in our study expected and experienced improved quality, duration and effectiveness of sleep:

"I am feeling better... At night, I could sleep when I had the nutrition...I am less worried and I could sleep quietly. "

Though experiencing tiredness and need for rests, some patients reported more energy and increasing muscle power due to HPN:

„I recognize that I am getting more power again... Today I can reach the shower cabin, sometimes I can do everything on my own. Sometimes I can towel myself at least. Before [starting HPN] I could not even get into the shower cabin. Now I can towel myself and then wait for my wife for further help.”

Of the component 'Body Structures,' structure of the stomach was the only category to be expected and to be experienced to improve. Of the component 'Activities and Participation', walking was the only category to be expected and to be experienced to improve. Arguably, this is to be seen in the context of increased energy and muscle power.

As described before, patients in stage 1 did not report eating and drinking as impaired, whereas patients in stage 2 did. In addition, only the patients in stage 2 experienced improvements in eating and drinking due to HPN. Eating and drinking can still be heavily limited in patients shortly after the start of HPN, as described frequently in relation to oral mucositis as a side effect from antineoplastic therapy [41].

### Relevant aspects that could not be expressed in ICF categories

Only few of the concepts extracted from the interviews could not be linked to specific ICF categories. Most relevant were aspects related to the ICF component 'Personal Factors', specifically aspects associated with coping strategies or spiritual meaningfulness of the situation. This is in line with the literature stating that cancer patients describe making sense of their situation and the development of coping skills as the most relevant issues [42,43].

### Methodological considerations

We have to point out that it was not the intention of our study (and of qualitative studies in general) to draw generalizing conclusions on the expectations and experiences towards functioning and health of cancer patients under HPN, or to report outcomes of HPN in various subgroups. Rather, the results of our study should provide a pool of patient-relevant items to be investigated in respect to prevalence and change over time in future studies.

Our study has a potential limitation. Selection of patients for the interviews could have been biased towards individuals with milder disease who would be ready to undergo an interview procedure. However, our findings have high face validity and are in line with the few studies conducted in this field. Thus, our study can contribute a first impression from the patients' perspective regardless of potential selection bias.

## Conclusions

The ICF proved to be a satisfactory framework to standardize the response of patients with cancer on HPN. For most aspects reported by the patients, a matching concept and ICF category could be found. However, the development of categories of the component 'Personal Factors' should be promoted to close the existing gap when analyzing interviews with the aim to explore the individuals' perspectives on functioning and health in specific situations. The identification and standardization of concepts derived from individual interviews was the first step towards creating new measures based on patients' preferences and experiences which both catch the most relevant aspects of functioning and are sensitive enough to monitor change associated to an intervention such as HPN in a vulnerable population with cancer.

## Competing interests

MM received a research grant by TravaCare Gmbh, Hallbergmoos, Germany. The sponsor contributed in the discussion regarding optimal study design and participant recruitment. The sponsor was not involved in collecting, analyzing and interpreting the data, in the writing of the manuscript, and in the decision to submit the manuscript for publication.

## Authors' contributions

MM and EG designed the study. MM carried out the interviews. MM and SL analyzed the data. All Authors interpreted the results and contributed in drafting the manuscript. All authors read and approved the final manuscript.

## Supplementary Material

Additional file 1 Interview guideline. Click here for file
