# Supplementary material for: Functioning and health in patients with cancer on home-parenteral nutrition: a qualitative study
Source: Health Qual Life Outcomes. 2010 Apr 16;8:41. doi: 10.1186/1477-7525-8-41 (PMC2862019; doi:10.1186/1477-7525-8-41)
Supplement: Additional file 1 — Interview guideline. [file 1477-7525-8-41-S1.DOC]

| **Entry question (stage 1&2 ):**  “Please tell me, how you feel in general.” | |
| --- | --- |
| **Questions on the ICF components (stage 1&2 ):** | |
| (1) “If you think about the functions of your body, your mind and your soul, what does not work the way it is supposed to?” (*Body Functions*) | |
| (2) “If you think about your body, in which parts are your problems?”( *Body Structures*) | |
| (3) “If you think about your daily life, what are your problems?” (*Activities and Participation*) | |
| (4) “If you think about your environment and your living conditions, what do you find helpful or supportive? If you think about your environment and your living conditions, what barriers do you experience?” (*Environmental Factors*) | |
| (5) “If you think about yourself, what is crucial when handling your current situation?” *(Personal Factors)* | |
| **Question on effects of HPE** | |
| stage 1: | Which aspects of functioning do you expect to improve due to the home-parenteral nutrition you undergo?” |
| stage 2: | Which aspects of functioning improved due to the home-parenteral nutrition you underwent?” |
